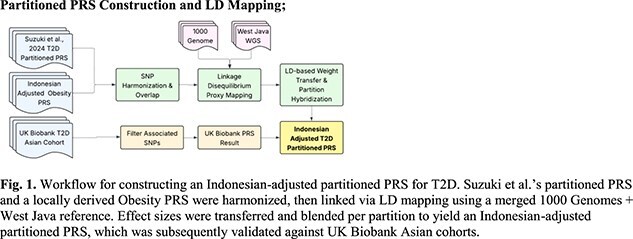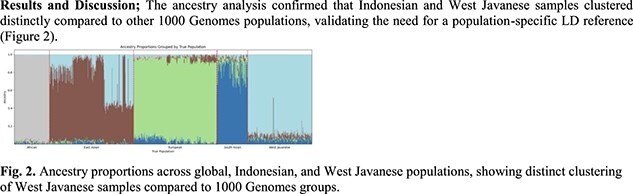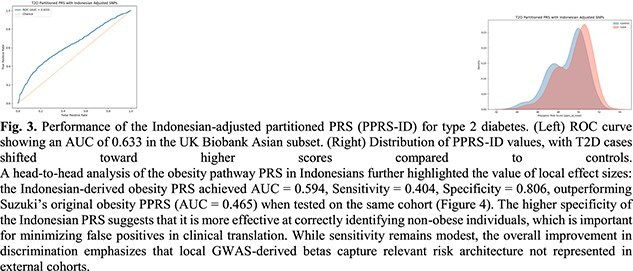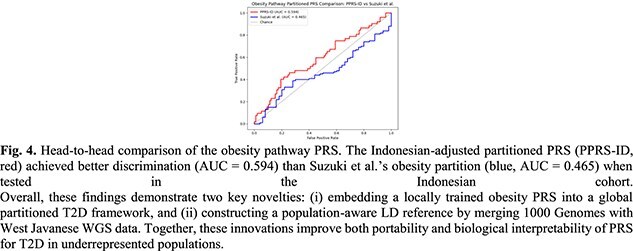# PPRS-ID: Indonesian-adjusted partitioned PRS for type 2 diabetes using obesity PRS integration and west Javanese population LD mapping

**DOI:** 10.1093/bib/bbaf631.011

**Published:** 2025-12-12

**Authors:** Kezia Irene, Jocelyn Verna Siswanto, Belinda Mutiara, Felicia Austin, Jonathan Susanto, Restu Unggul Kresnadi

**Affiliations:** Corporate Data Management, PT Kalbe Farma Tbk, 10510 Central Jakarta, Indonesia; Corporate Data Management, PT Kalbe Farma Tbk, 10510 Central Jakarta, Indonesia; Corporate Data Management, PT Kalbe Farma Tbk, 10510 Central Jakarta, Indonesia; Corporate Data Management, PT Kalbe Farma Tbk, 10510 Central Jakarta, Indonesia; Corporate Data Management, PT Kalbe Farma Tbk, 10510 Central Jakarta, Indonesia; Corporate Data Management, PT Kalbe Farma Tbk, 10510 Central Jakarta, Indonesia

## Abstract

**Background:**

Polygenic risk scores (PRS) are increasingly recognized as powerful tools for predicting complex diseases such as type 2 diabetes (T2D) [1,2]. However, their transferability across populations remains limited, especially for underrepresented groups such as Indonesians, due to differences in allele frequencies, linkage disequilibrium (LD) structures, and genetic architecture [3,4]. Suzuki et al. [5] recently introduced a multiancestry weighted partitioned PRS (PPRS) for T2D, which decompose genetic risk into biologically interpretable pathways, including obesity, body fat distribution, metabolic syndrome, and residual glycaemic regulation. Despite this innovation, no PPRS has yet been developed or validated in Southeast Asian populations.

In this study, we propose the Indonesian-adjusted partitioned PRS (PPRS-ID), which integrates an obesity PRS derived from an Indonesian cohort [6] with Suzuki’s partitioned T2D PRS framework. We further enhance transferability by applying LD proxy mapping using a novel merged reference panel that combines the 1000 Genomes Project [7] with whole-genome sequencing (WGS) data from West Javanese individuals [8]. This study presents the methodology, preliminary results, and a discussion of the implications for population-specific PRS development.

**Methods: Dataset:**

The Indonesian dataset consisted of 2936 individuals (959 males, 1977 females) recruited between 2021 and 2024, with body mass index (BMI) as the primary phenotype. Genotyping was performed using the KalGen01 custom SNP array, designed to capture both common and rare variants relevant to Indonesian populations. Standard quality control steps included call rate filtering, Hardy–Weinberg equilibrium testing, and relatedness checks.

To build a population-specific LD reference, we incorporated 227 whole-genome sequences (WGS) from West Javanese donors published by Ardiansyah et al [8]. These sequences provided population-specific haplotype structure and novel variant representation, complementing the 1000 Genomes reference.

For external evaluation, we used the UK Biobank Asian subset (n = 7901; 2690 with T2D and 5211 controls), consisting primarily of Chinese and other East Asian participants. BMI and T2D phenotype data were obtained from baseline assessments.

**West Javanese Data Processing:**

The whole-genome sequences from West Javanese donors conducted by previous studies [8], were first harmonized to the same reference assembly used for downstream analyses. The raw WGS VCFs (originally on GRCh37) were liftovered to GRCh38, then compressed, sorted, and partitioned into autosomal chromosomes (1–22) to enable chromosome-wise processing. We applied targeted QC/cleanup to remove problematic sites and genotype calls (for example, records with missing alternate alleles or inconsistent genotype encodings) so that variant representation would be compatible with the 1000 Genomes reference and with the partitioned PRS framework mapped on GRCh38.

To recover missing genotypes and produce a population-representative LD resource, we performed genotype imputation on each chromosome using the 1000 Genomes Project as the reference panel and an appropriate GRCh38 genetic map. Imputation filled sparse or missing calls in the West Javanese WGS while preserving local haplotype structure. Imputed West Java chromosomes were then merged with the corresponding 1000 Genomes chromosomes to create a combined GRCh38 reference panel. Each merged chromosome file was indexed and prepared for downstream analyses. The resulting 1000G + West-Java panel provided a harmonized, population-aware LD reference that we used for LD proxy mapping and for computing signed LD correlations when projecting T2D effect sizes onto Indonesian obesity SNPs.

**Partitioned PRS Construction and LD Mapping:**

An obesity PRS was first derived from the Indonesian cohort using genome-wide association analysis, using BMI as the continuous trait while also applying ancestry adjustment [5]. SNPs overlapping with Suzuki’s partitioned T2D PRS were harmonized on the GRCh38 build. Because overlap was limited, we expanded the SNP set via LD proxy mapping using a merged GRCh38 reference panel that included both the 1000 Genomes Project and WGS data from West Javanese individuals. Signed LD correlations (r) were computed within ±1 Mb windows, and effect sizes from T2D SNPs were projected onto obesity SNPs (β^^^ = r·β).

Partition-specific hybrid weights were then generated by combining projected T2D betas with obesity-derived betas according to biologically informed α parameters (α = 0.3 for obesity, α = 0.7 for residual glycaemic pathways, α = 0.3 for metabolic syndrome, α = 0.3 for body fat). PRS scoring was performed in PLINK2, and the resulting PPRS-ID was evaluated in the UK Biobank Asian subset.

**Results and Discussion:**

The ancestry analysis confirmed that Indonesian and West Javanese samples clustered distinctly compared to other 1000 Genomes populations, validating the need for a population-specific LD reference (Fig. 2).

We found 1 SNP overlap between Suzuki’s partitioned T2D SNPs and our Indonesian obesity PRS; however, the merged LD reference improved recovery of proxy SNPs across partitions by adding 10 matched SNPs with aligned R^2^ > 0.4. Evaluation of PPRS-ID in the UK Biobank Asian subset yielded an AUC of 0.633 for discriminating T2D cases from controls (Fig. 3). The distribution plots show a rightward shift in scores for cases compared to controls, demonstrating that transferability improves when pathway-specific weighting is combined with a locally trained component.

A head-to-head analysis of the obesity pathway PRS in Indonesians further highlighted the value of local effect sizes: the Indonesian-derived obesity PRS achieved AUC = 0.594, Sensitivity = 0.404, Specificity = 0.806, outperforming Suzuki’s original obesity PPRS (AUC = 0.465) when tested on the same cohort (Fig. 4). The higher specificity of the Indonesian PRS suggests that it is more effective at correctly identifying non-obese individuals, which is important for minimizing false positives in clinical translation. While sensitivity remains modest, the overall improvement in discrimination emphasizes that local GWAS-derived betas capture relevant risk architecture not represented in external cohorts.

Overall, these findings demonstrate two key novelties: (i) embedding a locally trained obesity PRS into a global partitioned T2D framework, and (ii) constructing a population-aware LD reference by merging 1000 Genomes with West Javanese WGS data. Together, these innovations improve both portability and biological interpretability of PRS for T2D in underrepresented populations.

**Conclusion:**

We present PPRS-ID, an Indonesian-adjusted partitioned PRS for T2D that integrates local obesity effect sizes with Suzuki’s PPRS architecture and leverages a merged 1000G + West Java LD reference. Preliminary results demonstrate moderate predictive accuracy (AUC 0.633 in UKBB Asians) and improved performance of the obesity partition in Indonesian data (AUC 0.594 versus 0.465). This study illustrates the feasibility of adapting partitioned PRS frameworks for underrepresented populations and underscores the importance of local genomic resources in precision medicine.

However, the current work is limited by the modest number of directly overlapping SNPs, reliance on proxy recovery via LD, and the relatively small size of available Indonesian and West Javanese datasets. These factors may constrain the stability and generalizability of effect size estimates. Future directions include validation in larger Southeast Asian cohorts, refinement of partition-specific weighting schemes, and exploration of clinical utility in personalized prevention and treatment strategies.

**References:**

1. Khera AV, Chaffin M, Aragam KG, et al. Genome-wide polygenic scores for common diseases identify individuals with risk equivalent to monogenic mutations. Nat Genet. 2018;50(9):1219–1224. https://doi.org/10.1038/s41588-018-0183-z

2. Torkamani A, Wineinger NE, Topol EJ. The personal and clinical utility of polygenic risk scores. Nat Rev Genet. 2018;19(9):581–590. https://doi.org/10.1038/s41576-018-0018-x

3. Duncan L, Shen H, Gelaye B, et al. Analysis of polygenic risk score usage and performance in diverse human populations. Nat Commun. 2019;10(1):3328. https://doi.org/10.1038/s41467-019-11,112-0

4. Martin AR, et al. Clinical use of current polygenic risk scores may exacerbate health disparities. Nat Genet. 2019;51(4):584–591. https://doi.org/10.1038/s41588-019-0379-x

5. Suzuki K, Hatzikotoulas K, et al. Genetic drivers of heterogeneity in type 2 diabetes pathophysiology. Nature. 2024;627(8003):347–357. doi:10.1038/s41586-024-07019-6.

6. Kalbe Farma Bioinformatics Division. Genome-wide association study of obesity in Indonesian cohort using the KalGen01 SNP array. (Unpublished, 2024).

7. 1000 Genomes Project Consortium, Auton A, Brooks LD, et al. A global reference for human genetic variation. Nature. 2015;526(7571):68–74. https://doi.org/10.1038/nature15393

8. Ardiansyah E, et al. Sequencing whole genomes of the West Javanese population in Indonesia reveals novel variants and improves imputation accuracy. Front Genet. 2025;15:1492602. https://doi.org/10.3389/fgene.2025.1492602